# Identification of subgroups of children in the Australian Autism Biobank using latent class analysis

**DOI:** 10.1186/s13034-023-00565-3

**Published:** 2023-02-20

**Authors:** Alicia Montgomery, Anne Masi, Andrew Whitehouse, Jeremy Veenstra-VanderWeele, Lauren Shuffrey, Mark D. Shen, Lisa Karlov, Mirko Uljarevic, Gail Alvares, Sue Woolfenden, Natalie Silove, Valsamma Eapen

**Affiliations:** 1 University of New South Wales, Sydney, Australia; 2 University of Western Australia, Perth, Australia; 3 Columbia University, New York, USA; 4 University of North Carolina, Chapel Hill, USA; 5 University of Melbourne, Melbourne, Australia; 6 Sydney Children’s Hospital Network, Randwick, Sydney, Australia

**Keywords:** Autism spectrum, Subgroups, Latent class analysis

## Abstract

**Background:**

The identification of reproducible subtypes within autistic populations is a priority research area in the context of neurodevelopment, to pave the way for identification of biomarkers and targeted treatment recommendations. Few previous studies have considered medical comorbidity alongside behavioural, cognitive, and psychiatric data in subgrouping analyses. This study sought to determine whether differing behavioural, cognitive, medical, and psychiatric profiles could be used to distinguish subgroups of children on the autism spectrum in the Australian Autism Biobank (AAB).

**Methods:**

Latent profile analysis was used to identify subgroups of children on the autism spectrum within the AAB (n = 1151), utilising data on social communication profiles and restricted, repetitive, and stereotyped behaviours (RRBs), in addition to their cognitive, medical, and psychiatric profiles.

**Results:**

Our study identified four subgroups of children on the autism spectrum with differing profiles of autism traits and associated comorbidities. Two subgroups had more severe clinical and cognitive phenotype, suggesting higher support needs. For the ‘Higher Support Needs with Prominent Language and Cognitive Challenges’ subgroup, social communication, language and cognitive challenges were prominent, with prominent sensory seeking behaviours. The ‘Higher Support Needs with Prominent Medical and Psychiatric and Comorbidity’ subgroup had the highest mean scores of challenges relating to social communication and RRBs, with the highest probability of medical and psychiatric comorbidity, and cognitive scores similar to the overall group mean. Individuals within the ‘Moderate Support Needs with Emotional Challenges’ subgroup, had moderate mean scores of core traits of autism, and the highest probability of depression and/or suicidality. A fourth subgroup contained individuals with fewer challenges across domains (the ‘Fewer Support Needs Group’).

**Limitations:**

Data utilised to identify subgroups within this study was cross-sectional as longitudinal data was not available.

**Conclusions:**

Our findings support the holistic appraisal of support needs for children on the autism spectrum, with assessment of the impact of co-occurring medical and psychiatric conditions in addition to core autism traits, adaptive functioning, and cognitive functioning. Replication of our analysis in other cohorts of children on the autism spectrum is warranted, to assess whether the subgroup structure we identified is applicable in a broader context beyond our specific dataset.

**Supplementary Information:**

The online version contains supplementary material available at 10.1186/s13034-023-00565-3.

## Background

Autism spectrum disorder is a common neurodevelopmental condition characterized by social and communication difficulties in the presence of restricted, repetitive, and stereotyped behaviours [1], with a prevalence of approximately 1% internationally [2]. Clinical, behavioural and biological heterogeneity are widely recognized as hallmark features of the autism spectrum (AS), and this heterogeneity poses a significant impediment to the identification of underlying aetiological processes and targeted treatment and support recommendations [3]. No single etiological pathway is anticipated to be able to explain the majority of the clinical or biological heterogeneity associated with the AS [4]. Rather, a myriad of aetiologies is proposed [5], and the effectiveness of differing treatment approaches will likely vary depending on the putative AS subtypes [6].

### Empirical approaches to subgroup identification in autistic populations

The identification of reproducible, valid subtypes within autistic populations is a priority research area in the context of neurodevelopment, to pave the way for identification of genetic and other biomarkers, and targeted treatment and support recommendations for this population [4]. It is encouraging to see that over time, the number of studies that have focused on characterizing potential ASD subgroups has increased and that emphasis has shifted from theoretically derived classifications of subtype to data-driven approaches [7]. A range of confirmatory and exploratory statistical approaches have been utilised for this purpose, such as different types of cluster analysis [5, 8], and latent class or profile analysis for cross-sectional and latent transition profile analysis for longitudinal data [9, 10]. These approaches all seek to identify similarities in patterns of observed data between individuals, and are therefore dependent upon the data variables selected for inclusion in the analysis [7]. The majority of previous studies that have used empirical methods to identify subgroups in autistic populations have classified individuals on the basis of behavioural traits (relating to social communication or RRBs, and occasionally traits indicative of psychiatric comorbidity e.g. anxiety), cognitive or adaptive function, or a combination of behavioural phenotype, cognition and adaptive function [11].

The most replicated findings from empirical studies of subgroup classification in autistic populations to date have yielded between two and four subgroups, defined in terms of a severity gradient (low, moderate, and high) [8–13], and/or two groups endorsing the DSM-5 diagnostic domains (social communication and interaction, and restricted, repetitive, and stereotyped behaviour) [7, 14–16]. Identified subgroups have not been consistently replicated across contexts, and have had limited prognostic value to date [17]. Sample size has been a limiting factor across many previously published studies, and use of summary outcome measures as indicator variables (composite scores reflecting categories of behaviour, e.g. total restricted, repetitive, and stereotyped behaviour), rather than measures of specific behaviours reflecting more nuanced phenotypic information. Overall summary scores conflate distinct subdomains that have different trajectories, differing associations with key demographic, cognitive and clinical variables and distinct underlying aetiology. By not examining individual phenotypic constructs, the ability to detect distinct subgroups (beyond a severity gradient) is greatly diminished. To delve beyond broad diagnostic categories with greater biological and prognostic relevance, constructs that represent specific core traits of autism, in addition to cognitive, medical, and psychiatric comorbidity, must be examined.

Co-occurring medical conditions are common in children with ASD and can significantly affect child and family functioning [18, 19], but few previous subtyping studies in autistic populations have used both core autism traits and data pertaining to significant comorbidities (such as seizures, gastrointestinal conditions, sleep disorders, and psychiatric conditions) as indicator variables [3]. Emerging findings suggest that comorbid conditions (sleep dysfunction, language impairment, immune dysfunction, gastrointestinal dysfunction, and seizures) may be important to discriminating between subgroups within autistic populations [20, 21].

In this study, we sought to empirically identify subgroups of children on the autism spectrum in the large, well-characterised, and nationally representative Australian Autism Biobank (AAB). We sought to do so on the basis of differing presentations of core traits of autism and co-occurring cognitive, medical, and psychiatric profiles. The AAB is a national data repository overseen by the Cooperative Research Centre for Living with Autism (Autism CRC) [22].

## Methods

Ethics to perform this study was granted by the University of New South Wales Human Research Ethics & Clinical Trials Governance Committee (HC190924). Access to phenotypic data for all children on the autism spectrum within the AAB (n = 1151), was obtained via the Autism CRC Utilisation Grant 1.073RU.

### Study sample

The AAB has previously been described in detail by Alvares et al. (2018) [22], and contains detailed phenotypic data and biological samples obtained from children (aged 2–17 years) on the autism spectrum, in addition to siblings, parents, and unrelated non-autistic controls. The empirical subgroup analysis performed in this study utilised detailed phenotypic data pertaining to children within the AAB with an autism spectrum diagnosis in accordance with DSM-IV or DSM-5 criteria [1], who were recruited between 2013 and 2018 across four sites in Perth, Brisbane, Sydney, and Melbourne.

Phenotypic data within the AAB was obtained from clinical assessments that utilised a range of administered measures and standardised questionnaires completed by parents or caregivers, including the Autism Diagnostic Observation Schedule-2 (ADOS-2) [23] or Autism Diagnostic Observation Schedule-G (ADOS-G) [24], the Developmental, Dimensional and Diagnostic Interview (3di) [25], Vineland Adaptive Behaviour Scale-II [26], and the Short Sensory Profile-2 (SSP-2) [27]. Cognitive functioning was assessed using the Mullen Scales of Early Learning (MSEL) for those aged below six years [28], or Wechsler Intelligence Scale for Children 4th edition (WISC-IV) for those above 6 years of age [29]. Morphometric measures (height, weight, head circumference), and detailed child and family medical history, were collected for all participants [22]. Data coverage varies across measures, and in this study, latent profile analysis was conducted within the subset of *n* = 754 children on the AS within the AAB for whom the deepest phenotypic data (obtained using the 3di standardised parental autism interview) was available. All standardized assessments were administered by raters without knowledge of cytokine measurements.

### Variables

In this study, indicator variables pertaining to the core autism traits and psychiatric comorbidity were based on data obtained using the 3di, a standardised parental interview [25]. To reflect aspects of phenotype associated with DSM-5 category A criteria (describing persistent differences in social communication and social interaction), composite-based scores generated by the 3di were used to obtain three continuous measures of difficulty associated with social-emotional reciprocity, non-verbal communication, and development and maintenance of relationships. A further 11 composite-based scores generated by the 3di were used as indicator variables to represent restricted, repetitive, and stereotyped behaviours associated with autism. Indicator variables selected to represent aspects of phenotype pertaining to comorbid psychiatric, behavioural, cognitive, and medical conditions were chosen on the basis of existing evidence in the literature for their relevance in relation to autism phenotype [2], and on the basis of their availability in the AAB. Accordingly, 37 indicator variables were selected to represent co-occurring cognitive, behavioural, psychiatric and medical aspects of phenotypes. Further details regarding the variables utilised in this study are available in Additional File 1.

### Statistical analyses

Latent class analysis (LCA) and latent profile analysis (LPA) are empirical methods of identifying underlying subgroups (often termed classes) within a dataset based on patterns of data across categorical variables, or continuous variables (or a mixture of both), respectively [30]. In this study, latent profile analysis was conducted using 37 indicator variables, describing 14 core traits of autism, and 23 aspects of phenotype across cognitive, psychiatric, behavioural, medical, and morphometric domains. Continuous variables were standardised to z scores prior to analysis. The objective of the analysis was to identify the model that best describes the latent structure within the dataset, starting with a one-class model and then fitting successive models with increasing numbers of classes. Models were estimated using maximum likelihood estimation with robust standard errors, such that there are several solutions around which a model can converge (local maxima). To ensure that a global maximum was identified, we ran at least 200 starts and 20 iterations for each model solution. Optimal profile solution was derived based on the specific goodness of fit statistics and interpretability. These statistics included the loglikelihood ratio, with higher values supporting models of better fit, and the Bayesian Information Criterion (BIC) and Akaike Information Criterion (AIC), with smaller values supporting models of better fit and parsimony [31]. The entropy statistic ranges from 0 to 1, with values closer to 1 reflecting better classification accuracy of individuals into classes depending on their model-based posterior probabilities [32]. Finally, the Lo-Mendell-Rubin Adjusted Likelihood Ratio Test (LMR-LRT) was used to compare models with different numbers of classes, with a non-significant value suggesting that a model with one fewer class is a better explanation of the data [33]. LPA yields predicted probabilities of class membership, and cases were assigned to their most likely class based on these probabilities. Mean scores of continuous indicator variables and differing probabilities for categorical variables were examined by class, in addition to age and gender. Latent profile analysis was performed in Mplus Version 8.6, and all other aspects of the statistical analysis were performed in SPSS Version 26.

## Results

The overall AAB cohort had a mean age of 7.5 ± 3.9 years, and was predominantly male (78.2%). Deep phenotypic data (obtained from the 3di Developmental, Dimensional and Diagnostic Interview [25]) was available for *n* = 754 participants, who were selected for use in the latent profile analysis. These children had similar demographic profiles to those in the overall AAB cohort [Table 1]. Cohort characteristics are described in Table 2.

**Table 1:** Demographics

	Australian Autism Biobank Cohort			
	All children on the autism spectrum		Full phenotypic data available^a^	
*N*	1151		754	
Child characteristics				
Age in years				
Mean (SD)	7.5 (3.9)		7.5 (3.8)	
Range	1.9–20.9		2.1–20.9	
Sex (*n*)				
Male	900	78.2%	595	78.9%
Female	251	21.8%	159	21.1%
Maternal ethnicity (*n*)				
Caucasian	755	65.6%	514	68.2%
Aboriginal	7	0.6%	6	0.8%
Asian	94	8.2%	68	9.0%
Maori/Pacific				
Islander	11	1.0%	8	1.1%
Other	74	6.4%	54	7.2%
Missing	210	18.2%	104	13.8%
Paternal ethnicity (*n*)				
Caucasian	763	66.3%	525	69.6%
Aboriginal	9	0.8%	4	0.5%
Asian	85	7.4%	58	7.7%
Maori/Pacific				
Islander	10	0.9%	8	1.1%
Other	65	5.6%	45	6.0%
Missing	219	19.0%	114	15.1%
Overall level of autism-spectrum related symptoms^b^				
High	385	33.5%	259	34.4%
Moderate	559	48.5%	374	49.6%
Low	125	10.9%	74	9.8%
Minimal to Nil	30	2.6%	10	1.3%
Missing	52	4.5%	37	4.9%

^a^Defined on the basis of Developmental, Dimensional and Diagnostic Interview (3di) data availability

^b^Defined by the Autism Diagnostic Observation Schedule ADOS-2 Comparison Score

**Table 2:** Cohort characteristics

Developmental, dimensional and diagnostic interview (3di) Scores of Core Autism Traits	(Mean, SD)	
Difficulties with social-emotional reciprocity (range 0–2)	1.0 (0.3)	
Difficulties with non-verbal social communication (range 0–2)	0.9 (0.3)	
Difficulties with developing and maintaining relationships (range 0–2)	1.0 (0.3)	
Stereotyped and repetitive speech (range 0–45)	15.8 (10.5)	
Stereotyped movements (range 0–9)	3.0 (2.3)	
Stereotyped use of objects (range 0–9)	3.8 (2.5)	
Adherence to routines (range 0–15)	6.1 (4.0)	
Ritualised patterns of behaviour (range 0–15)	6.2 (4.0)	
Resistance to change (range 0–12)	4.4 (3.4)	
Restricted and fixated interests (range 0–27)	11.0 (6.0)	
Sensory interests (range 0–15)	5.0 (3.5)	
Hyposensitivity to sensory input (range 0–9)	2.5 (2.3)	
Auditory hypersensitivity (range 0–4)	2.2 (1.6)	
Other sensory hypersensitivity (range 0–24)	8.9 (5.9)	
Other characteristics	(Mean, SD)	
Overall intellectual ability (percentile)	25.1 (29.0)	
Head circumference (z score)	− 0.5 (1.2)	
3di inattentiveness score (Range 0–9)	2.9 (2.3)	
3di hyperactivity and impulsivity score (range 0–9)	3.2 (2.4)	
3di child communication checklist fluency of speech score	28.8 (5.2)	
Vineland adaptive behaviour scale-II adaptive composite score (percentile)	15.4 (22.6)	
	n	%
History of language delay	462	61.3%
History of gross motor delay	242	32.1%
History of regression	259	34.4%
Co-occurring anxiety disorder	168	22.3%
History of depression and/or suicidality	73	9.7%
History of tics	98	13.0%
History of hallucinations	62	8.2%
Co-occurring oppositional defiant or conduct disorder	90	11.9%
History of self-injurious behaviour	64	8.5%
History of seizure(s)	77	10.2%
Sleep onset difficulties	179	23.7%
Sleep maintenance difficulties	104	13.8%
History of gastrointestinal dysfunction	309	41.0%
Birthweight		
Low	66	10.5%
Normal	481	76.3%
Macrosomic	83	13.2%
Food allergy (Acute reaction)	72	9.5%
Food allergy (Non-acute reaction)	150	19.9%
Non-food allergy	179	23.7%
Hyperextensibility	83	11.0%

### Latent profile analysis

**Fig. 1:**
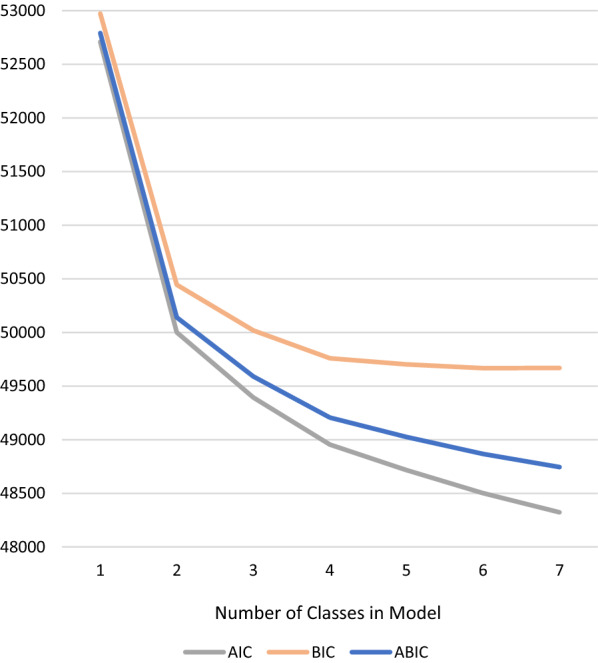
Scree plot containing latent class fit statistics for children on the autism spectrum in the Australian Autism Biobank

Latent profile analysis of 37 indicator variables describing 14 core traits of autism and 23 other aspects of phenotype yielded a best-fitting model with four-classes. Table 3 displays goodness of fit indices for the latent profile analysis. With each addition of one class to the model, the BIC and adjusted BIC values decreased, but plateaued after the four-class model [Fig. 1], whilst the LMR-LRT test suggested that the four-class model did not provide significantly better fit than the three-class model (p = 0.122) [Table 3]. Across models, entropy values were greater than 0.85, suggesting good precision of latent classifications.

**Table 3:** Latent class fit statistics for children on the autism spectrum in the Australian Autism Biobank

Classes	Loglikelihood	Starts replicated	Free parameters	AIC^a^	BIC^b^	ABIC^c^	LMR-LRT^d^ (p)	Entropy
1	− 26297.881	200 20	57	52709.76	52973.41	52792.41	N/A	N/A
2	− 24904.636	200 20	96	50001.27	50445.31	50140.47	< 0.0001	0.90
3	− 24561.991	200 20	135	49393.98	50018.41	49589.73	< 0.0001	0.87
4	− 24303.256	200 20	174	48954.51	49759.33	49206.81	0.1216	0.88
5	− 24145.339	200 20	213	48716.68	49701.89	49025.53	0.5549	0.87
6	− 23998.863	200 20	252	48501.73	49667.32	48867.12	0.7722	0.87
7	− 23870.439	800 80	291	48322.88	49668.87	48744.84	0.4666	0.87

^**a**^Akaike information criterion

^**b**^Bayesian information criterion

^**c**^Sample adjusted bayesian information criterion

^**d**^Lo-Mendell-Rubin Likelihood Ratio Test

Based on goodness of fit statistics, both the three- and four-class models were further considered with the focus on understanding whether addition of the fourth profile provided clinically meaningful information over the three-profile solution. The three-class model described subgroups that are best characterized by the severity gradient across measures of core autism traits, medical comorbidities, and psychiatric comorbidities rather than showing distinct clinical profiles. The four-class model was deemed to be more substantively meaningful and showed unique patterns across specific clinical subdomains rather than being only distinguished by overall severity. Identified profiles were characterized as a ‘Fewer Support Needs Group,’ ‘Higher Support Needs with Prominent Language and Cognitive Challenges Group,’ ‘Moderate Support Needs with Emotional Challenges Group’ and a ‘Higher Support Needs with Prominent Medical and Psychiatric and Comorbidity Group’ [Table 4]. Notable differences between subgroups identified in the 4-class model are summarised in Table 4.

**Table 4:** Characteristics by latent class for children on the autism spectrum in the Australian Autism Biobank: four class model

	Class one		Class two		Class three		Class four		Overall	
*N*	220		83		259		192		754	
Age (y), mean (SD)	6.5 (3.5)		5.9 (3.2)		8.1 (4.0)		8.4 (3.9)		7.5 (3.8)	
Sex (*n*)										
Male	172	78.2%	72	86.7%	207	79.9%	144	75.0%	595	78.9%
Female	48	21.8%	11	13.3%	52	20.1%	48	25.0%	159	21.1%
Developmental, dimensional and diagnostic interview (3di) scores of core autism traits (mean, SD)										
Difficulties with social-emotional reciprocity (range 0–2)	0.8 (0.3)		1.4 (0.2)		0.9 (0.2)		1.2 (0.3)		1.0 (0.3)	
Difficulties with non-verbal social communication (range 0–2)	0.8 (0.3)		1.2 (0.2)		0.8 (0.2)		1.1 (0.3)		0.9 (0.3)	
Difficulties with developing and maintaining relationships (range 0–2)	0.9 (0.3)		1.3 (0.3)		0.9 (0.3)		1.1 (0.2)		1.0 (0.3)	
Stereotyped and repetitive speech (range 0–45)	11.4 (9.9)		8.3 (11.8)		17.7 (8.3)		21.4 (9.0)		15.8 (10.5)	
Stereotyped movements(range 0–9)	1.5 (1.4)		3.7 (2.3)		2.7 (1.9)		4.8 (2.3)		3.0 (2.3)	
Stereotyped use of objects (range 0–9)	2.0 (1.6)		3.3 (2.1)		3.7 (1.9)		6.3 (2.0)		3.8 (2.5)	
Adherence to routines (range 0–15)	2.4 (1.7)		3.4 (2.5)		6.6 (2.6)		10.9 (2.6)		6.1 (4.0)	
Ritualised patterns of behaviour (range 0–15)	3.2 (3.1)		4.9 (3.3)		6.0 (2.6)		10.6 (2.6)		6.2 (4.0)	
Resistance to change (range 0–12)	1.4 (1.3)		2.5 (2.2)		4.4 (2.2)		8.6 (2.4)		4.4 (3.4)	
Restricted and fixated interests (range 0–27)	6.2 (4.7)		10.7 (5.6)		11.4 (4.6)		16.0 (5.0)		11.0 (6.0)	
Sensory interests (range 0–15)	2.5 (2.3)		7.0 (3.1)		4.4 (2.6)		7.7 (3.5)		5.0 (3.5)	
Hyposensitivity to sensory input (range 0–9)	1.5 (1.6)		2.9 (2.4)		2.2 (2.0)		3.7 (2.8)		2.5 (2.3)	
Auditory hypersensitivity (range 0–4)	1.6 (1.6)		1.6 (1.5)		2.4 (1.6)		2.9 (1.5)		2.2 (1.6)	
Other sensory hypersensitivity (range 0–24)	4.7 (4.2)		6.8 (4.5)		10.0 (5.4)		13.1 (5.5)		8.9 (5.9)	
Other characteristics (mean, SD)										
Overall intellectual ability (percentile)	21.9 (28.0)		6.6 (19.9)		31.2 (30.3)		27.3 (27.9)		25.1 (29.0)	
Head circumference (z score)	− 0.5 (1.3)		− 0.9 (1.3)		− 0.3 (1.2)		− 0.5 (1.2)		− 0.5 (1.2)	
Inattentiveness(range 0–9)	1.5 (1.8)		3.7 (1.8)		3.0 (2.2)		4.0 (2.3)		2.9 (2.3)	
Hyperactivity and impulsivity (range 0–9)	1.8 (1.7)		3.2 (1.8)		3.4 (2.4)		4.5 (2.5)		3.2 (2.4)	
Fluency of speech score	29.0 (5.4)		26.5 (5.0)		29.8 (5.2)		28.2 (4.9)		28.8 (5.2)	
Adaptive composite score (percentile)	21.7 (27.1)		2.3 (4.6)		18.8 (22.8)		9.9 (18.0)		15.4 (22.6)	
Categorical characteristics (probabilities)										
History of regression	0.2807		0.6208		0.3058		0.4683		0.344	
History of seizure(s)	0.0702		0.1035		0.1115		0.1638		0.102	
Sleep onset difficulties	0.1339		0.2162		0.3085		0.3372		0.237	
Sleep maintenance difficulties	0.0875		0.2281		0.0915		0.2642		0.138	
Language delay	0.5963		0.8936		0.5247		0.6320		0.613	
Gross motor delay	0.4668		0.4035		0.2038		0.2775		0.321	
Gastrointestinal dysfunction	0.2239		0.4502		0.4391		0.5700		0.410	
Food allergy (likely IgE mediated)	0.0319		0.0591		0.1135		0.1626		0.095	
Food allergy (non-acute reaction)	0.0765		0.1857		0.2220		0.3176		0.199	
Non-food allergy	0.0947		0.1081		0.2843		0.4016		0.237	
Anxiety disorder	0.0199		0.1670		0.2791		0.4110		0.223	
Birthweight										
Low	0.0947		0.1594		0.0830		0.1227		0.1048	
Normal	0.8343		0.6812		0.7118		0.7975		0.7635	
Macrosomic	0.0710		0.1594		0.2052		0.0798		0.1317	
Oppositional defiant or conduct disorder	0.0303		0.0171		0.1681		0.2055		0.119	
Hyperextensibility	0.0942		0.1284		0.0974		0.1372		0.110	
History of self-injurious behaviour	0.0168		0.1514		0.0947		0.1209		0.085	
History of tics	0.0660		0.1288		0.1608		0.1641		0.130	
History of depression and/or suicidality	0.0615		0.0188		0.1317		0.1274		0.097	
History of hallucinations	0.0271		0.0176		0.0953		0.1905		0.082	

In this study, Class 1 (29.2%) described a ‘Fewer Support Needs Subgroup,’ with fewer social communication difficulties and fewer restricted, repetitive and stereotyped behaviours than the overall group, with higher levels of adaptive functioning. This subgroup was somewhat more likely to have had delayed acquisition of early gross motor milestones than the overall group, but were less likely to have experienced developmental regression, and had lower likelihood of cognitive, psychiatric, and medical comorbidity, compared to the overall group Table 5

**Table 5:** Summary of subgroup differences for children on the autism spectrum in the Australian Autism Biobank, based on four-class latent profile model

	Class one: fewer support needs group	Class two: higher support needs with prominent language and cognitive challenges group	Class three: moderate support needs with emotional challenges group	Class four: higher support needs with prominent medical and psychiatric and comorbidity group
Social Communication Difficulties	Mean subgroup levels of difficulty **below** overall group mean scores	Mean subgroup levels of difficulty **above** overall group mean, and most different from group mean overall	Mean subgroup levels of difficulty similar to overall group mean	Mean levels of difficulty **above** overall group mean scores
Restricted, Repetitive, Stereotyped Behaviours	Average levels of difficulty **below** group mean across all RRB categories	Sensory seeking and hyposensitivity subgroup scores **above** overall group mean. Repetitive speech, insistence on sameness, ritualistic and routine-focused behaviour, and sensory sensitivity scores **below** overall group mean scores	Subgroup mean similar to overall group mean across all RRB categories	Mean scores **above** overall group mean scores across all RRB categories
Cognitive ability	Subgroup mean similar to overall group mean	Mean subgroup cognitive ability **below** overall group mean, and most different overall	Subgroup mean similar to overall group mean	Subgroup mean similar to overall group mean
Adaptive Functioning	Subgroup mean **above** overall group mean	Subgroup mean **below** overall group mean and most different overall	Subgroup mean similar to overall group mean	Subgroup mean **below** overall group mean
Regression	Subgroup probability of regression **lower** than overall group	**Highest** probability of regression	Subgroup probability of regression similar to overall group	Subgroup probability of regression **higher** than overall group
Language	Subgroup probability of language delay similar to overall group	**Highest** probability of language delay	Subgroup probability of language delay similar to overall group	Subgroup probability of language delay similar to overall group
Motor	Subgroup probability of motor delay **higher** than overall group	Subgroup probability of motor delay **higher** than overall group	Subgroup probability of motor delay **lower** than overall group	Subgroup probability of motor delay similar to overall group
Medical comorbidity	Subgroup probability of seizures, gastrointestinal dysfunction, allergy **lower** than overall group	Subgroup probability of seizures, gastrointestinal dysfunction, allergy, similar to overall group	Subgroup probability of seizures, gastrointestinal dysfunction, allergy similar to overall group	Subgroup probability of seizures, gastrointestinal dysfunction, allergy **higher** than overall group
Psychiatric comorbidity	**Lowest** probability of anxiety and ADHD, with probability of depression, defiance, hallucinations, and tics **lower** than in overall group	**Lowest** probability of defiance and depression, with probability of anxiety **lower** than in overall group but scores of inattention **higher** than in overall group	**Highest** probability of depression, with probability of defiance **higher** than in overall group	**Highest** probability of anxiety, ADHD, defiance and hallucinations, with subgroup probability of depression and tics **higher** than in overall group
Sleep	**Lowest** probability of sleep onset and maintenance difficulties	Probability of sleep onset difficulties similar to overall group with **higher** probability of sleep maintenance difficulties	Probability of sleep onset difficulties **higher** but probability of sleep maintenance difficulties **lower** than overall group	**Highest** probability of sleep onset and maintenance difficulties
Self-injurious behaviour (SIB)	Subgroup probability of SIB **lower** than overall group	**Highest** probability of SIB	Subgroup probability of SIB similar to overall group	Subgroup probability of SIB **higher** than overall group

Subgroup differences in phenotype (compared to overall group) are highlighted in bold. Phenotypic characteristics that were most different in one subgroup are underlined

Class 2 (11.0%) described a ‘Higher Support Needs with Prominent Language and Cognitive Challenges Subgroup,’ with the greatest social communication and cognitive difficulties overall. This subgroup had the highest probability of regression, language delay, and self-injurious behaviour. Compared to the overall group, this subgroup had higher mean scores for sensory seeking behaviours, and lower mean scores for all other RRBs (including sensory aversive behaviours, repetitive behaviours, fixations, routine-focused behaviours and insistence on sameness). This subgroup had a similar probability of seizures, gastrointestinal dysfunction, and allergy, compared to the overall group, but had a higher probability of sleep maintenance difficulties.

Class 3 (34.4%) described a ‘Moderate Support Needs with Emotional Challenges Subgroup,’ that had similar mean scores of core autism traits, cognitive ability, and adaptive functioning, to the overall group. This group had the highest probability of experiencing depression and/or suicidality, and had a higher probability of exhibiting sleep onset difficulties and defiant behaviours than the overall group.

Finally, Class 4 (25.5%) described a ‘Higher Support Needs with Prominent Medical and Psychiatric Comorbidity Subgroup.’ This subgroup had the highest amount of social communication difficulties and the highest scores of restricted, repetitive and stereotyped behaviours overall. Their mean scores of cognitive ability were similar to the overall group, but with lower levels of adaptive functioning. This subgroup had the highest probabilities of medical comorbidity, sleep dysfunction, and psychiatric comorbidity.

## Discussion

This latent profile analysis identified four subgroups within the AAB that were distinguished not solely on the basis of an overall severity gradient, but on differing profiles in relation to core autism traits and associated comorbidities. Class 2 and Class 4 both described subgroups of children with higher mean scores of social communication difficulty than the overall group, but Class 2 had the highest probability of language delay and lowest mean cognitive scores, highest scores of sensory seeking behaviour, with lower scores of all other RRBs compared to the overall group. For children within Class 2, social communication challenges, language delay, and cognitive impairment appear to be prominent features of the neurodevelopmental profile, with sensory seeking behaviours but otherwise less prominent RRBs. Class 2 had the lowest probability of depression and a lower probability of anxiety than children in Class 4, who also had high support needs (in relation to core autism traits, medical comorbidity, and psychiatric comorbidity), with cognitive scores similar to the overall group mean. Finally, children in Class 3 had mean scores that were similar to the overall group mean for measure of core autism traits, cognitive ability, and adaptive functioning, but had the highest probability of experiencing depression and/or suicidality.

Comparison of findings reported between empirical subtyping studies in autistic populations is complicated by significant diversity in the range of variables utilised to construct subgroups. The strengths of this study include deep phenotyping encompassing the comprehensive range of behavioural, cognitive, medical, and psychiatric variables that were utilised in our subtyping analysis and sample size that afforded good power to detect distinct subgroups. In a recent systematic review of published subtyping studies in autistic populations, of the 156 identified studies, only 16% had a sample size greater than N = 1000 [34]. Studies varied significantly in relation to sample size (ranging between N = 17 and N = 20658), statistical methods, and indicator variables selected to define subtypes. The median number of variables utilised to conduct subtyping analyses was 20, with 80% of studies including fewer than 20 variables overall. The majority of studies utilised core autism traits to construct subtypes, with only a minority incorporating medical aspects of comorbidity into their analysis. Four previous studies included a combination of behavioural, cognitive, psychiatric, and medical indicator variables [7, 10, 20, 35], and an additional two studies performed empirical subgrouping analysis among children on the autism spectrum using sleep-related [36] or immune-related [37] variables only. Our findings are most amenable to comparison with the four previous studies that utilised behavioural, cognitive, psychiatric, and medical indicator variables for subgrouping analyses, and these are explored in greater detail below.

Wiggins et al. performed latent class analysis in a similarly sized sample of 707 children on the autism spectrum, and incorporated variables reflecting a similar range of behavioural, cognitive, psychiatric, and medical aspects of phenotype, to those used in this study, although standardised measures used to reflect these differed [7]. Four subgroups were identified, including a subgroup characterised by mild language delay with cognitive rigidity, another with mild language and motor delay with dysregulation, another with general developmental delay, and another with significant delay with repetitive motor behaviours [7]. Notable parallels were observed between these previously identified subgroups [7], and those identified in this study. Most notably, both studies identified a subgroup characterised by mean cognitive scores in the average range, with high rates of psychiatric and medical comorbidity including gastrointestinal complaints, sleep dysfunction, and seizures. Both studies identified two subgroups with mild and moderate challenges across most variables, and a subgroup primarily characterised by lowest mean scores of cognitive ability. However, some differences between our findings were also apparent. Although both analyses yielded a subgroup with mild social communication difficulties and comorbidity overall, our study did not replicate associated increased scores of cognitive rigidity in this subgroup, as was observed by Wiggins et al. [7]. Secondly, the subgroup with the highest degree of cognitive impairment in the study by Wiggins et al. were at greatest risk of seizures and had high scores for motor mannerisms, whereas in our study the subgroup with the lowest mean cognitive score had low mean scores across all RRBs, with the notable exception of sensory seeking.

More limited comparison is possible between our findings and those reported in other empirical subgrouping studies in autistic populations, even among other studies that examined medical comorbidity, due to differences in the overall range of variables utilised. Veatch et al. performed hierarchical clustering using variables representing core autism traits, adaptive functioning, age, and head circumference, but did not include other aspects of psychiatric or medical comorbidity in their analysis [10]. Their analysis identified two subgroups characterised by lower and higher severity across measures. As in our study, differing patterns of RRBs were found to be more useful for discriminating between subgroups than were scores of social communication, and head circumference did not significantly vary between subgroups [10].

Another previous study that used a range of behavioural, cognitive, psychiatric, and medical aspects of phenotype performed *k*-means clustering in a cohort of 3,278 children on the AS [35]. Three subgroups were identified, including one predominantly characterised by high rates of co-occurring psychiatric and medical comorbidity (particularly immune-related conditions and gastrointestinal dysfunction), one predominantly characterised by cognitive delay and highest probability of seizures, and one predominantly characterised by low scores of difficulty across measures [35]. As was observed in our study, the subgroup with highest rates of psychiatric and (non-epileptic) medical comorbidity had mean cognitive abilities similar to the overall group mean [35].

Medical aspects of comorbidity have previously been important in distinguishing between subgroups of children on the AS using hierarchical clustering and *k*-means [20]. Four subgroups were identified, including one characterised by prominent immune abnormalities accompanied by some circadian and sensory issues, one with prominent circadian and sensory dysfunction, one with prominent stereotypies, and one with prominent cognitive challenges and disruptive behaviour [20]. The subgroup with prominent immune-related dysfunction (e.g. allergy, atopy, autoimmunity) demonstrated the lowest probability of cognitive impairment, with higher probability of obstetric complications and gastrointestinal disturbance, compared to the other subgroups and overall cohort [20]. Our findings did not replicate this pattern of medical comorbidity across subgroups. Rather, in our study medical comorbidity was most prominent in the subgroup of children with the highest scores of difficulty associated with core autism traits and psychiatric comorbidity, and the probability of medical comorbidity was similar to the overall cohort among the group with prominent cognitive and language challenges.

Future opportunities for research will explore additional validation methods of the four subgroups identified in our LPA, as outlined in the recently proposed framework for subgroup validation, named the SUbtyping Validation Checklist (SUVAC) [34]. Cross-method replication will be explored within the AAB using alternative empirical subtyping methods, and replication will also be explored using a second Australian dataset. Subgroup differences in overall adaptive functioning (based on the ABC score from the VAB-3) provided external evidence of meaningful clinical differences between the subgroups identified in our study, since adaptive functioning was not used as an indicator variable in our LPA. Future opportunities for research will also explore parallel validation of the subgroups we identified, involving use of a second set of indicator variables that reflect similar aspects of phenotype to those used in our initial LPA, to assess whether identified subgroups cluster in a similar substantive manner.

Beyond replication, identification of subgroups among children with AS will facilitate targeted, individualized treatment recommendations and identification of biological associations that may not be apparent when treating the heterogenous overall population as one cohort. In turn, we will seek to identify which subgroups of children with ASD are most likely to benefit from specific intervention options, and to better understand the varying aetiological pathways relevant to autism.

## Conclusion

Our study identified four subgroups within the AAB that were distinguished not solely on the basis of a ‘support needs gradient’, but on differing profiles in relation to core autism traits and associated comorbidities. Individuals within subgroups share greater homogeneity in relation to their phenotype presentations than the group overall, and may have greater similarity in terms of shared aetiology and response to treatments. Our findings highlight the importance of including co-occurring medical, psychiatric, and cognitive aspects of phenotype among the indicator variables utilised in subgrouping analyses in autistic populations. Further replication studies are warranted for validation of the subgroups identified in our analysis, including longitudinal follow-up studies to explore stability over time and prognosis.

## Supplementary Information

**Additional file 1**: **Table S1**: Indicator Variables Describing Core Autism Traits based on 3di Data. **Table S2**: Indicator Variables Describing Comorbid Conditions.

## Data Availability

The data utilised within this study is available via direct application to the Australian Autism Biobank.
