# Supplementary material for: Identification of subgroups of children in the Australian Autism Biobank using latent class analysis
Source: Child Adolesc Psychiatry Ment Health. 2023 Feb 20;17:27. doi: 10.1186/s13034-023-00565-3 (PMC9940381; doi:10.1186/s13034-023-00565-3)
Supplement: Supplementary file 1 — Additional file 1: Table S1: Indicator Variables Describing Core Autism Traits based on 3di Data. Table S2: Indicator Variables Describing Comorbid Conditions. [file 13034_2023_565_MOESM1_ESM.docx]

**Additional file Table 1: Indicator Variables Describing Core Autism Traits based on 3di Data**

| Indicator Variable | Underlying Phenotypic Construct | Contributing 3di Items (#) |
| --- | --- | --- |
| Difficulties with Social-Emotional Reciprocity | Unusual social approach | 237,716,717,1073 |
|  | Difficulties with age appropriate social behaviour | 317,321,322,323,677,728 |
|  | Difficulty with back and forth conversation | 682,688,705,706,719,744,747,1142 |
|  | Reduced sharing of interests | 303 |
|  | Reduced sharing of emotions and response to emotion | 223,224,304,305,307-310,313,314, 624,625 |
|  | Reduced offering to share | 297,298,299,300,301 |
| Difficulties with Non-verbal Social Communication | Difficulties reading facial expressions | 226,707 |
|  | Reduced facial expressiveness | 257,258,259-264,708 |
|  | Reduced social smiling | 249,251,252,711 |
|  | Reduced eye contact | 248,709,710 |
|  | Reduced use and reading of body language | 271,272,278,279,280,281,282,283,284,285,729,737,742 |
|  | Reduced imitation | 751,752 |
|  | Reduced appropriateness of non-verbal interaction | 743 |
|  | Reduced appropriateness of spontaneous emotions | 269,741 |
| Difficulties with Developing and Maintaining Relationships | Difficulty adjusting behaviour to suit social contexts | 318,319,320,676,702,703,704,720,1227 |
|  | Reduced imaginative play with peers | 361,362,363,364,369,370,371,372 |
|  | Reduced cooperative social play | 330,331,332,333,360,655 |
|  | Difficulties making friends | 345,347,349,350,351,352,353,355,713,726,727 |
| Stereotyped and repetitive speech | Repetitive conversation or vocalisation, idiosyncratic language, echolalia, neologism | 678,679,680,689-697,748,749,1221 |
| Stereotyped movements | Repetitive movements of the hands, fingers, or body | 766,767,982 |
| Stereotyped use of objects | Lining up toys, repetitive ways of viewing video content | 755,976,1219 |
| Adherence to routines | Routine focused behaviour, difficulties with transitioning | 979,1209,1210,1211,1213 |
| Ritualised patterns of behaviour | Rituals involving actions, verbal rituals, play rituals | 340,750,756,1216,1218 |
| Resistance to change | Insistence on sameness | 758,1212,1214,1215 |
| Restricted and fixated interests | Preoccupations and unusual interests | 721,722,723,754,973,974,975, 1208,1287 |
| Sensory interests | Unusual interests of a tactile, olfactory, oral, or visual nature | 757,977,978,1220,1288 |
| Hyposensitivity to sensory input | Decreased sensitivity to pain or temperature | 1293,1294,1295 |
| Auditory hypersensitivity | Increased sensitivity to ordinary sounds or music | 99,101,103,105,107,109,111 |
| Other Sensory Hypersensitivity | To dietary or tactile textures, taste, visual or olfactory stimuli | 1217,1230,1277,1278,1289, 1290,1291,1292 |

**Additional file Table 2: Indicator Variables Describing Comorbid Conditions**

| Domain | Indicator Variable | Data Source | Variable Type | |
| --- | --- | --- | --- | --- |
| **Cognition** | **Overall Intellectual Ability**  (Percentile) | Overall Composite Score or Full-Scale IQ (FSIQ) from the MSEL^1^ or WISC-IV^2^ | | Continuous |
| **Developmental** Language | **Language Delay**  Delayed acquisition of language milestones | 3di^3^ | | Dichotomous Categorical |
|  | **Articulation** | 3di^3^ CCC^4^ subscale A - fluency of speech score | | Continuous |
| Motor | **Gross Motor Delay**  Delayed onset of sitting unsupported and/or walking | 3di^3^ | | Dichotomous Categorical |
| Regression | **Regression**  History of loss of physical or language skills | 3di^3^ | | Dichotomous Categorical |
| **Psychiatric and Behavioural** | **Inattentiveness**  Composite-based score | 3di^3^ | | Continuous |
|  | **Hyperactivity and Impulsivity**  Composite-based score | 3di^3^ | | Continuous |
|  | **Anxiety**  Co-occurring anxiety disorder e.g. generalised anxiety, separation anxiety agoraphobia, phobia, panic | 3di^3^ | | Dichotomous Categorical |
|  | **Depression and/or Suicidality** Co-occurring depression and/or history of suicidality | 3di^3^ | | Dichotomous Categorical |
|  | **Tics**  History of motor or vocal tics | 3di^3^ | | Dichotomous Categorical |
|  | **History of hallucinations**  Possible or definite visual or auditory hallucinations | 3di^3^ | | Dichotomous Categorical |
|  | **Oppositional Defiant and/or Conduct Disorder**  Co-occurring oppositional defiant or conduct disorder | 3di^3^ | | Dichotomous Categorical |
|  | **Self-Injurious Behaviour**  Definite or severe e.g. biting, hair pulling, head banging | 3di^3^ | | Dichotomous Categorical |
| **Medical** | **Birthweight Category**  Low <2500g  Normal 2500-4000g  Macrosomia >4000g | Family History Questionnaire – Participant Medical History | | Categorical |
|  | **Seizures**  Any previous history of seizures or fits | Family History Questionnaire – Participant Medical History | | Dichotomous Categorical |
|  | **Sleep Onset Difficulties**  Requires longer than 20 minutes to falls asleep - Rarely or sometimes, versus usually (5-7 times per week) | Family History Questionnaire – Children's Sleep Habits | | Dichotomous Categorical |
|  | **Sleep Maintenance Difficulties -** Wakes more than once a night - Rarely or sometimes, versus usually (5-7 times per week) | Family History Questionnaire – Children's Sleep Habits | | Dichotomous Categorical |
|  | **Gastrointestinal Dysfunction**  History of consulting a health professional in relation to constipation, diarrhoea, reflux, vomiting, or abdominal complaints | Family History Questionnaire – Participant Medical History | | Dichotomous Categorical |
|  | **Food Allergy (Likely IgE mediated, acute reaction) –** e.g. respiratory symptoms, angioedema, vomiting, hives, loss of consciousness | Family History Questionnaire – Participant Medical History | | Dichotomous Categorical |
|  | **Food Allergy (Likely non-IgE mediated) –** e.g. non-acute gastrointestinal dysfunction, irritability or other symptoms | Family History Questionnaire – Participant Medical History | | Dichotomous Categorical |
|  | **Non-Food Allergy –** History of reactions to non-food allergens | Family History Questionnaire – Participant Medical History | | Dichotomous Categorical |
|  | **Hyperextensibility**  Of finger or thumb joints | 3di^3^ | | Dichotomous Categorical |
| **Morphometric** | **Head circumference**  **(z score)** | Clinical Proforma Form | |  |

^1^ Mullen Scales of Early Learning (MSEL) [27]

^2^ Wechsler Intelligence Scale for Children 4th edition (WISC-IV) [28]

^3^ Developmental, Dimensional and Diagnostic Interview (3di) [24]

^4^ Children’s Communication Checklist – 2^nd^ Edition (CCC-2) [37]
